# Acceptable Postvoid Residual Urine Volume after Vaginal Delivery and Its Association with Various Obstetric Parameters

**DOI:** 10.1155/2018/5971795

**Published:** 2018-08-06

**Authors:** Wen Sze Choe, Beng Kwang Ng, Ixora Kamisan Atan, Pei Shan Lim

**Affiliations:** Department of Obstetrics and Gynaecology, UKM Medical Centre, Kuala Lumpur, Malaysia

## Abstract

**Background:**

Urinary retention and voiding dysfunction is a distressing event and relatively common in immediate postpartum period. This study aims at investigating the range of postvoid residual urine volume after vaginal delivery and its association with various obstetric parameters.

**Methods:**

This was a prospective observational study of women who delivered vaginally in Universiti Kebangsaan Malaysia Medical Centre from March 2017 to September 2017. Those who were able to void within 6 hours after delivery, the voided volume measurements were taken at their second void followed by measurement of residual urine using a transabdominal ultrasound scan. For those unable to void at 6 hours postpartum, the bladder volume was measured. If the bladder volume was 500 ml or more, an indwelling catheter would be inserted and kept for 24 hours.

**Results:**

A total of 155 patients who fulfilled the inclusion were recruited. There were 143 (92.3%) patients who had residual urine volume of less than 150 ml at second void. Out of these 143 patients, 138 (96.5%) had residual urine volume of less than 100 ml, and among the 138 patients, 119 (86.2%) had residual urine volume of less than 50 ml. The median residual urine volume was 10 ml (2, 42). The overall rate of postpartum urinary retention (PPUR) was 7.7%; 6 (3.85%) had overt retention and 6 (3.85%) had covert retention. Primiparity, duration of active phase of labour, duration of second stage of labour, epidural analgesia, episiotomy, instrumental delivery, and perineal pain score were independent risk factors associated with postpartum urinary retention.

**Conclusion:**

Postpartum urinary retention complicates approximately 7.7% of vaginal deliveries. Majority (86.2%) of them had residual urine volume less than 50 ml. Obstetrics factors independently associated with PPUR include primiparity, duration of active phase of labour, duration of second stage of labour, epidural analgesia, episiotomy, instrumental delivery, and degree of perineal pain.

## 1. Introduction

Urinary retention and voiding dysfunction are distressing events and relatively common in immediate postpartum period. Voiding dysfunction is departure from normal sensation or function, experienced by the woman during or following the act of micturition [1]. However, the exact incidence of postpartum urinary retention (PPUR) is uncertain and varies considerably between 0.2% and 37.0% [2–5]. These vast differences may be due to different definition used to define PPUR, as well as the different diagnostic criteria and modalities used in different studies.

Up till now, there is no local database in Malaysia or a standard literature definition for PPUR. Various definitions for a significant postvoid residual (PVR) urine volume have been published by different authors, ranging from 40 to 500 ml [6]. Based on published literature, PPUR was defined as an “absence of spontaneous micturition after six hours of vaginal delivery” or “no spontaneous micturition after removal of indwelling catheter after lower segment caesarean section” [7]. International Continence Society revised the definition of urinary retention as inability to pass urine despite persistent effort [1]. Yip et al. further classified PPUR as overt or covert urinary retention [7]. Overt retention refers to the inability to void in the presence of signs and symptoms of urinary retention. On the contrary, covert retention refers to women that are asymptomatic of urinary retention, with a postvoid residual bladder volume of more than 150 ml, identified by ultrasound screening or by catheterization [3, 7, 8].

The pathophysiology of PPUR is unclear, but likely to be multifactorial, involving physiological, neurological, and mechanical changes that occur during pregnancy, labour, and postpartum. An elevated progesterone level in pregnancy and the immediate postpartum period result in reduced smooth muscle tone, leading to a dilated bladder, ureters, and renal pelvis. Together with decreased bladder sensation and tone, these may cause hypotonic bladder in the early puerperium, and the bladder is less sensitive to filling hence can withstand more distention [4]. All postpartum women that deliver vaginally are prone to PPUR because of the perineal denervation that may occur. It is difficult to predict which patient will develop PPUR. However, certain patients with high risk factors can be identified, such as those who received intrapartum epidural analgesia, had instrumental deliveries, had prolonged first or/and second stage of labour, were primigravida, had macrosomic babies, or received augmentation with oxytocin during labour [3, 4, 8]. Thus, the aim of this study was to investigate the range of postvoid residual urine volume after vaginal delivery and its association with various obstetric parameters.

## 2. Materials and Methods

### 2.1. Study Design

This was a prospective observational study of women who delivered vaginally in Universiti Kebangsaan Malaysia Medical Centre (UKMMC) from March 2017 to September 2017. All women with vaginal delivery to a single infant were included. Exclusion criteria were women with preexisting voiding dysfunction before pregnancy such as detrusor underactivity or outflow obstruction (urethra), those who underwent caesarean section, had multiple gestation or presentation other than vertex, those who required in-dwelling catheterization intrapartum or postpartum for reasons other than acute urinary retention such as severe preeclampsia, obstetric anal sphincter injury, and postpartum hemorrhage, and women taking medicines likely to affect ability to void spontaneously.

### 2.2. Procedure

Approval to conduct this study was obtained from the UKM Medical Research Ethic Committee and given the project code FF-2017-079. There was no funding granted for this study.

Patients who delivered in UKMMC and fulfilled the inclusion criteria were approached in postnatal ward within six hours postpartum. Study information sheets were distributed and informed consent was taken.

Demographic data such as age, parity, body mass index (BMI), and gestation at delivery were recorded. Intrapartum details including duration of active phase of labour and second stage of labour, birth weight of baby, use of oxytocin for augmentation and its duration, epidural analgesia, mode of delivery, episiotomy, any extended perineal tear, or vulva haematoma were recorded as well.

Previously, various methods have been used to assess PVR, which include the use of transabdominal, transvaginal, or translabial ultrasound or catheterization. Automated system now allows a more simplified PVR estimation by abdominal ultrasound. Although catheterization is the gold standard for PVR measurement, this may cause discomfort to the patient; it is invasive and carries the risk of introducing infection and trauma to the urethra. Therefore, ultrasonography is the method of choice for its safety, convenience, and efficiency, making its use beneficial in a wide variety of populations [6, 9–11]. Griffith et al. showed that the bladder volume estimated by transabdominal ultrasound had an accuracy of 0.84 with repeatability of measurement ±10.2% [12]. The amount of the postvoid residual urine volume in the bladder was calculated using the prolate ellipsoid method described in Dicuio et al. [11]:

(1) $$\begin{array}{c} \text{volume}=D1\times D2\times D3\times 0.52, \end{array}$$

where *D*1 = widest diameter in the transverse scan, *D*2 = anterior-posterior diameter in the longitudinal scan, and *D*3 = cephalocaudal diameter in the longitudinal scan.

Those who were able to void within 6 hours after delivery, their voided volume measurements were then taken at their second void using a measuring cup. Residual urine was then measured using transabdominal ultrasound scan within 10 minutes after voiding. The perineal pain score was assessed at the same time with the measurement of bladder volume using the numeric rating scale of 0 to 10.

For those unable to void at 6 hours, bladder volume was measured using transabodminal ultrasound scan. Patients with bladder volume of 500 ml or more were then catheterized and an indwelling catheter would be kept for 24 hours followed by a trial of void. The catheterized urine volume was also recorded. For patients with a bladder volume of less than 500 ml, a repeat scan would be done at two hours or immediately after void, whichever earlier.

### 2.3. Statistical Analysis

All data in the checklist was collected in an electronic database and analyzed using IBM SPSS statistics for Windows, Version 23.0® (Armonk, NY:IBM Corp). A descriptive study was used to summarize the demographic characteristics and the range of postvoid residual urine volume. The median (Quartile) value was used when a normal distribution was absent. Qualitative variables are given as number (percentage). Association of various obstetric parameters with postvoid residual urine volume were analyzed using chi-square (*χ*^2^) and Mann–Whitney *U* test/Fisher exact test. Pearson correlation was used to assess the relationship between various obstetrics factors with the volume of residual urine.

## 3. Results

**Figure 1:**
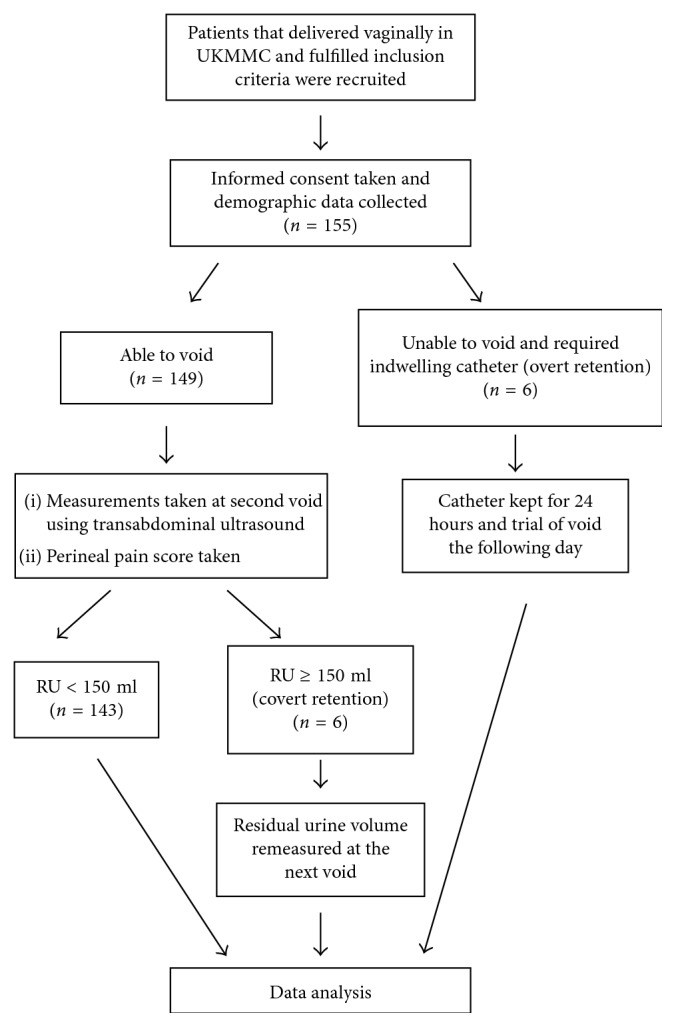
Flow chart.

A total of 163 patients were approached during the study duration, but eight patients declined to participate in the study. Thus, a total of 155 patients who delivered vaginally at UKMMC were recruited (Figure 1). The patients' median age was 30.0 (27.0, 34.0) years old and body mass index was 26.5 (24.3, 31.0) kg/m^2^ at delivery. Less than half of the participants were primiparous (35%). There were no significant differences between women with RU ≥ 150 ml and RU < 150 ml (Table 1).

**Figure 2:**
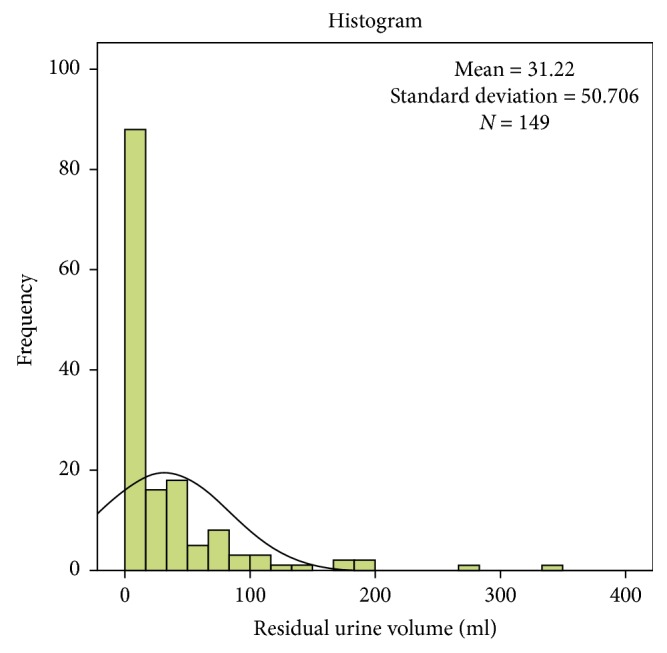
Postvoid residual urine volume.

In this study population of 155 patients, 143 (92.3%) patients had residual a urine volume of less than 150 ml. Out of these 143 patients, 138 (96.5%) had a residual urine volume of less than 100 ml, and the majority (86.2%) had a residual urine volume of less than 50 ml. The median residual urine volume was 10 ml (2.0, 42.0) (Figure 2), whereas the voided volume ranged from 30 ml to 900 ml.

The overall incidence of postpartum urinary retention was 7.7%: 6 (3.85%) had overt retention, requiring catheterization and 6 (3.85%) had covert retention. Among the six patients that had covert retention, the range of voided urine at second void was 150 ml to 550 ml, and the residual urine measured ranged from 173 to 338 ml. These patients remained asymptomatic despite residual urine of more than 150 ml. Subsequent measurement of residual urine volume upon discharge was less than 150 ml.

There were six patients that had overt retention requiring indwelling catheter. Majority of patients with overt retention were primiparous with baby's birth weight ranging from 3.0 kg to 3.5 kg. All of them had episiotomy with four out of six of them having instrumental delivery. Five out of six patients with overt retention had indwelling catheter for only one day, while one required catheterization for up to one week due to failed trial of void, which was case no 1 who had the highest catheterized urine volume of 1200 ml. Case 6 could only void minimally but no symptoms of suprapubic fullness or pain. Ultrasound scan revealed a bladder volume of 650 ml; therefore, an indwelling catheter was inserted (Table 2).

Residual urine of ≥150 ml was taken as a cutoff value for postpartum urinary retention (PPUR) as 92.3% of our study population had RU < 150 ml. This study population had a median birth weight of 3060 g. The duration of labour was significantly longer in those with PPUR compared to those with no PPUR. Median duration of the active phase of labour was 375 min versus 135 min (*p*=0.001), and median duration of the second stage was 30 min versus 8 min (*p*=0.007). Primiparity (*p*=0.001), those who received epidural analgesia (*p*=0.004), those who had episiotomy (*p*<0.001), and those who had instrumental delivery (*p*=0.004) were significantly associated with higher rate of PPUR. However, use of oxytocin augmentation or duration of epidural analgesia did not show any significant association with PPUR. There was a significant difference of postpartum perineal pain score between those with PPUR and those with no PPUR (*p*<0.001) (Table 3).

There was significant positive correlation of duration of active phase of labour (*r*=0.23, *p*=0.005), duration of the second stage of labour (*r*=0.306, *p*=0.001), perineal pain score (*r*=0.272, *p*=0.001), and intrapartum blood loss (*r*=0.202, *p*=0.013) with residual urine volume. The body mass index had significant negative correlation (*r*=−0.216, *p*=0.08) with residual urine volume (Table 4).

## 4. Discussion

There are numerous studies on symptomatic postpartum urinary retention with consensus on various diagnostic definitions. However, we tend to overlook those postpartum women with high postvoid residual urine volume (PVR) but remained asymptomatic. Up until today, there are no standard definitions for a significant PVR. Various studies showed a wide range of 40 to 500 ml [6]. Currently, there is no national guideline available in Malaysia. This could be a pilot study to stimulate further work towards formation of a national guideline. The aim of this study is to find out the range of PVR in women after vaginal delivery and to identify its associated risk factors. This can help obstetricians to individualize more appropriate bladder care after delivery.

In our study population, 92.3% had postvoid residual urine volume of <150 ml, and the similar cutoff value was taken as postpartum urinary retention by many authors [3, 7, 8]. The incidence of postpartum urinary retention was 7.7%, which is similar with previous studies such as the study by Cavkaytar et al. that reported an incidence of 8.1% [2]. This was contradicting with the study done by Ajenifuja et al. in which the prevalence of PPUR in their study population was 29.4% and very much higher than that of ours [8]. This could be due to the methodological variation; despite that they were using the same cutoff value of 150 ml as diagnosis of PPUR.

In this study, the asymptomatic women had a wide range of postpartum PVR, varied from 0 to 338 ml. Of those patients with PVR ≥ 150 ml, six (3.35%) remained asymptomatic (covert) with PVR ranging from 173 to 338 ml. In a recent study done by Mulder et al. among 745 women who delivered vaginally, 347 (47%) were diagnosed with covert PPUR (PVR ≥ 150 ml), of whom 26% had PVR more than 250 ml (75th percentile) [13]. This showed that many women might not exhibit any clinical symptoms of urinary retention despite high PVR. Fortunately, subsequent measurement of PVR on these six women with covert retention showed PVR of less than 150 ml, which was consistent with the study by Yip et al. which showed women with covert retention would return to normal within four days [7].

The exact pathophysiology of high postpartum PVR remained unclear. Most of the literature studied on the association of various obstetric parameters with the risk of PPUR. Maternal characteristics are difficult to compare, as very often they were not mentioned in these studies. Our study did not show any significant difference with regard to maternal age, BMI, and gestational age at delivery with the risk of PPUR. This was consistent with the finding by Cavkaytar et al. [2]. Similar to Carley et al.[14] and Buchanan and Beckmann [15], we observed a higher PVR value in primigravidas. This may be due to relatively longer duration of labour and higher incidence of episiotomy in primigravidas. There was 90.9% of primigravidas who had episiotomy performed in our study population with median of duration of active phase of labour and second stage of 235 minutes and 20 minutes, respectively.

The effect of intrapartum epidural analgesia on the risk of PPUR is controversial. Liang et al. demonstrated women with spontaneous vaginal delivery who received epidural analgesia had significant longer duration of labour and higher postpartum residual volume [16]. Many other literatures showed significant association of PPUR with epidural [2, 3, 8, 17–20]. Regional analgesia causes temporary disruption of afferent inputs to the spinal and pontine micturition centre. This inhibits the reflex mechanism that normally induces micturition. Subsequently, the bladder may become overdistended, reducing the contractile ability of the bladder [4, 21]. Nevertheless, this can be influenced by other confounding obstetric factors. On the contrary, Weissman et al. showed a weaker relationship between epidural and PPUR, with no significant difference of PVR comparing patients with or without epidural in labour [21].

Instrumental delivery, prolonged labour, and episiotomy may impair postpartum voiding in two ways, neurologically or mechanically. Neurologically, instrumental delivery might damage the pudendal nerves that affect the reflexes and voluntary mechanism in normal micturition. Similarly, perineal trauma might result in mechanical obstruction due to vaginal haematoma or oedema [4]. Higher incidence of postpartum urinary retention in this group was well reported in many studies [2, 7, 22–24]. These risk factors were significantly associated with higher PVR in our study. This is contradicting to the findings by Kekre et al. in which 87.7% patients had episiotomy but did not have significant association with PPUR [3]. However, they demonstrated that instrumental delivery and prolonged labour were significant risk factors for PPUR [3]. Fortunately, there was no extended perineal trauma or vaginal haematoma in our study population.

We also noted a significant correlation of postpartum perineal pain score with PVR whereby a higher pain score was associated with higher PVR. Urethral overactivity may ensue from pain, thus giving rise to functional obstruction [25]. Many of the studies did not address this parameter. This is important as we can provide adequate postpartum analgesia to reduce PVR that may further reduce the risk of postpartum urinary retention.

## 5. Limitations

Several limitations were identified. Firstly, the sample size was small, which may limit our ability to identify the actual incidence of PPUR. A larger sample size would also illustrate a better understanding of the associated obstetric parameters. Secondly, our study did not look into the long-term sequelae of those with high residual urine volume.

## 6. Conclusion

Postpartum urinary retention complicates approximately 7.7% of vaginal deliveries. Obstetrics factors independently associated with PPUR include primiparity, duration of active phase of labour, duration of second stage of labour, epidural analgesia, episiotomy, instrumental delivery, and degree of perineal pain.

## Figures and Tables

**Table 1:** Social demographic characteristics between women with RU ≥ 150 ml and RU < 150 ml.

Characteristics	Total (*n*=155)	RU ≥ 150 ml (*n*=12)	RU < 150 ml (*n*=143)	*p*
Maternal age, years	30.0 (27.0, 34.0)	29.5 (26.2, 32.8)	30.0 (27.0, 34.0)	0.479
Educational level, *n* (%)				
** **(1) Primary	1 (0.6)	0	1 (0.7)	0.752
** **(2) Secondary	66 (42.6)	4 (33.3)	62 (43.4)	
** **(3) Tertiary	88 (56.8)	8 (66.7)	80 (55.9)	
Body mass index (kg/m^2^)	26.5 (24.3, 31.0)	26.4 (23.4, 28.9)	26.5 (24.3, 31.0)	0.553
Last childbirth 5 years and less, *n* (%)	141 (91.0)	12 (100)	129 (90.2)	0.603

All parameters are expressed in median (quartile) unless specified.

**Table 2:** Summary of patients that had overt retention requiring indwelling catheter.

Case	Para	Gestational age	Birth weight (g)	Duration of active phase/second stage (min)	Use of oxytocin	Use of epidural	Instrument delivery	Episiotomy	Symptoms of AUR	Catheterized urine volume (ml)	RU at trial of void after off CBD (ml)
1	1	39	3020	540/55	Yes	Yes	No	Yes	Suprapubic fullness and pain	1200	50
2	1	39 + 5	3350	110/64	No	No	Yes	Yes	Suprapubic fullness	500	18
3	1	37 + 4	3060	330/9	No	Yes	Yes	Yes	Suprapubic fullness and pain	500	22
4	2	38 + 3	3140	150/16	No	No	No	Yes	Suprapubic fullness and pain	500	130
5	1	39 + 6	3560	600/23	Yes	Yes	No	Yes	Suprapubic fullness and pain	500	50
6	2	38	3360	420/2	No	Yes	No	Yes	—	600	160

**Table 3:** Obstetrics parameters between study group and control group.

Characteristics	Total (*n*=155)	RU ≥ 150 ml (*n*=12)	RU < 150 ml (*n*=143)	*p*
Primiparity, *n* (%)	55 (35.5)	10 (83.3)	45 (31.5)	0.001
Gestational age at delivery	39.2 (38.1, 40.0)	39.2 (38.1, 40.0)	39.2 (38.1, 40.0)	0.727
Duration of active phase of labour, minutes	150 (75, 240)	375 (152, 525)	135 (60, 240)	0.001
Duration of second stage, minutes	9 (5,18)	30 (9.5, 53.8)	8 (5, 17)	0.007
Oxytocin augmentation, *n* (%)	50 (32.3)	6 (50.0)	44 (30.8)	0.203
Duration of oxytocin augmentation, hours	3.5 (1.5, 6.0)	5.5 (2.9, 6.8)	3.2 (1.5, 5.9)	0.142
Epidural analgesia, *n* (%)	17 (11.0)	5 (41.7)	12 (8.4)	0.004
Duration of epidural analgesia, hours	5.0 (3.7, 7.0)	5.0 (4.5, 7.3)	5.5 (2.4, 7.0)	0.422
Episiotomy, *n* (%)	74 (47.7)	12 (100)	62 (43.4)	<0.001
Instrumental delivery	17 (11.0)	5 (41.7)	12 (8.4)	0.004
Type of instrumental delivery, *n* (%)				
** **(1) Vacuum	16 (94.1)	4 (80.0)	12 (100.0)	0.294
** **(2) Forceps	1 (5.9)	1 (20.0)	0	
Birth weight, grams	3060 (2800, 3270)	3170 (3030, 3357)	3050 (2770, 3270)	0.098
Estimated blood loss, ml	300 (200, 300)	300 (300, 300)	300 (200, 300)	0.017
Perineal pain score	2.0 (1.0, 3.0)	3.5 (3.0, 5.8)	2 (1.0, 3,0)	<0.001

All expressed in median (quartile) unless specified.

**Table 4:** Correlation between various obstetric parameters with residual urine volume.

Obstetric parameters	*r*	*p* value
Maternal age	−0.136	0.098
Body mass index	−0.216	0.008
Gestational age at delivery	0.008	0.924
Duration of active phase of labour	0.230	0.005
Duration of second stage of labour	0.306	<0.001
Duration of oxytocin augmentation	0.134	0.364
Duration of epidural analgesia	0.143	0.640
Interval from delivery to first void	0.058	0.48
Interval of first void and second void	−0.115	0.16
Perineal pain score	0.272	0.001
Birth weight	−0.072	0.380
Blood loss	0.202	0.013

Pearson correlation *r* test: *r* ≤ 0.3: weak correlation; 0.3 < *r* < 0.7: moderate correlation; *r* ≥ 0.7: strong correlation.
